# Heat-Stress and Light-Stress Induce Different Cellular Pathologies in the Symbiotic Dinoflagellate during Coral Bleaching

**DOI:** 10.1371/journal.pone.0077173

**Published:** 2013-12-04

**Authors:** C. A. Downs, Kathleen E. McDougall, Cheryl M. Woodley, John E. Fauth, Robert H. Richmond, Ariel Kushmaro, Stuart W. Gibb, Yossi Loya, Gary K. Ostrander, Esti Kramarsky-Winter

**Affiliations:** 1 Office of Public Health Studies, John A. Burns School of Medicine, University of Hawaii – Manoa, Honolulu, Hawaii, United States of America; 2 Pacific Biosciences Research Center, University of Hawaii, University of Hawaii – Manoa, Honolulu, Hawaii, United States of America; 3 Haereticus Environmental Laboratory, Clifford, Virginia, United States of America; 4 Environmental Research Institute, North Highland College, UHI Millennium Institute, Thurso, Scotland, United Kingdom; 5 National Oceanic & Atmospheric Administration, National Ocean Service, Center for Coastal Environmental Health and Biomolecular Research, Charleston, South Carolina, United States of America; 6 Department of Biology, University of Central Florida, Orlando, Florida, United States of America; 7 Kewalo Marine Laboratory, Pacific Biosciences Research Center, University of Hawaii, Honolulu, Hawaii, United States of America; 8 The National Institute for Biotechnology and the Department of Biotechnology Engineering, Ben Gurion University, Beer Sheva, Israel; 9 Department of Zoology, George S. Wise Faculty of Life Sciences, Tel Aviv University, Tel Aviv, Israel; 10 Office for the Vice President for Research. Florida State University, Tallahassee, Florida, United State of America; King Abdullah University of Science and Technology, Saudi Arabia

## Abstract

Coral bleaching is a significant contributor to the worldwide degradation of coral reefs and is indicative of the termination of symbiosis between the coral host and its symbiotic algae (dinoflagellate; *Symbiodinium* sp. complex), usually by expulsion or xenophagy (symbiophagy) of its dinoflagellates. Herein, we provide evidence that during the earliest stages of environmentally induced bleaching, heat stress and light stress generate distinctly different pathomorphological changes in the chloroplasts, while a combined heat- and light-stress exposure induces both pathomorphologies; suggesting that these stressors act on the dinoflagellate by different mechanisms. Within the first 48 hours of a heat stress (32°C) under low-light conditions, heat stress induced decomposition of thylakoid structures before observation of extensive oxidative damage; thus it is the disorganization of the thylakoids that creates the conditions allowing photo-oxidative-stress. Conversely, during the first 48 hours of a light stress (2007 µmoles m^−2^ s^−1^ PAR) at 25°C, condensation or fusion of multiple thylakoid lamellae occurred coincidently with levels of oxidative damage products, implying that photo-oxidative stress causes the structural membrane damage within the chloroplasts. Exposure to combined heat- and light-stresses induced both pathomorphologies, confirming that these stressors acted on the dinoflagellate via different mechanisms. Within 72 hours of exposure to heat and/or light stresses, homeostatic processes (e.g., heat-shock protein and anti-oxidant enzyme response) were evident in the remaining intact dinoflagellates, regardless of the initiating stressor. Understanding the sequence of events during bleaching when triggered by different environmental stressors is important for predicting both severity and consequences of coral bleaching.

## Introduction

Coral bleaching is a physiological phenomenon in which the symbiosis between the coral host and its symbiotic dinoflagellate terminates [1]. As a result of environmental stressors, bleaching events can increase coral susceptibility to infectious diseases, reduction in reproductive fitness, and can eventually lead to the collapse of coral reef ecosystems [2]–[4]. Field observations of coral bleaching were first described in 1914 [5], but it was not until 1925 that Boschma [6] provided evidence that the coral's symbiotic dinoflagellates were digested by the host animal. Yonge [7] and Yonge and Nicholls [8] challenged this theory by arguing that the symbiotic dinoflagellates were expelled from the endoderm of the cnidarian, and not digested. Their expulsion theory was corroborated to occur in sea anemones by Smith [9], and went unchallenged until the work of Steele and Goreau [10], who reasserted that dinoflagellates were digested. In subsequent years, strong evidence for *in situ* degradation of dinoflagellates was demonstrated by a number of workers, both as a function of normal physiology and bleaching [1], [11]–[14]. Recent evidence substantiates expulsion as a mechanism of bleaching [15]–[17]. Thus, coral bleaching may result from a number of non-exclusive mechanisms, including host-cell detachment, *Vibrio* infection, viral-induced lysis of zooxanthellae, and zooxanthella programmed-cell-death [18]–[21], though the trigger(s) for the initiating these processes, as well as the processes themselves, remain elusive.

In recent decades, studies of bleaching predominantly focused on what happens to the symbiotic dinoflagellate (aka, zooxanthella) during a bleaching event, and whether dissociation of the symbiosis initiates by the dinoflagellate symbiont or by its host. For example, *in hospite* degradation of the dinoflagellate occurs in several coral species during natural (field) high-temperature or high-light events, either via self-induced dinoflagellate degradation or host xenophagy [11]–[13]. Dunn and co-workers [20] argued that in sea anemones, algal programmed-cell-death may be a prominent mechanism by which symbiotic dinoflagellates degrade, but their methodology did not sufficiently distinguish between a general necrotic response and programmed-cell-death. In corals, symbiotic dinoflagellates can induce several cellular acclimatory defenses that correlate with increased tolerance to bleaching-associated stress. These defenses include induction of mycosporine-like amino acids, heat-shock proteins, anti-oxidant enzymes and compatible solutes, and changes in photosynthetic accessory pigments [22]–[25]. Induction of reactive oxygen species, accumulation of oxidative damage products, and degradation of Photosystem II also have been correlated with many environmental inducers of bleaching, such as heat stress and light stress [23], [24], [26], [27]. To date the specific role each stressor plays during bleaching is unclear, and their exact mechanism(s) of action and time-sequence of occurrence remains unknown [24], [28], [29].

Field observations of sudden-onset solar bleaching (high-light-induced bleaching) in the coral *Goniastrea aspera* indicated that dinoflagellate loss resulted from *in hospite* algal degradation; gastrodermal cells hosting dinoflagellates exhibited progressive degradation of the dinoflagellates; and perplexing behaviors of chlorophyll *a* and *c* concentrations occurred during the bleaching process [13]. Subsequent investigation examining the west-east bleaching behavior of *G. aspera* inhabiting tidal-flats in Phuket, Thailand, showed the importance of the host's physiological processes as a factor in bleaching [23]. Shallow-water colonies exposed to high light during seasonally low tides had higher concentrations of host antioxidant enzymes and heat shock proteins than polyps on less-exposed sides, and therefore lost fewer symbiotic algae [23]. However, the investigators were unable to deduce the role the symbiotic dinoflagellates played in either initiating bleaching or possibly tolerance to bleaching [23], adding to the controversy concerning the dinoflagellate as a determinant of bleaching [1], [14], [28], [29]. Recent work from our laboratories indicates that during a bleaching event, symbiotic dinoflagellates are digested by the coral host using an autophagy-associated pathway termed symbiophagy [30]. It remains unclear if the dinoflagellate is affected during the initial phase of bleaching, before symbiophagy is morphologically evident in the holobiont. To address this question, the morphological and cellular responses occurring in the *in hospite* dinoflagellate symbiont of the coral *Pocillopora damicornis*, were characterized by subjecting them to acute exposures of heat-stress and light-stress and combined stress bleaching conditions over a period of five days. Changes in the dinoflagellates' cellular structural-integrity, accumulation of oxidative damage-products, heat-shock protein and anti-oxidant protein responses, as well as photosynthetic pigments were examined in depth, in the early stages of the experimentally induced stress.

## Materials and Methods

### Coral collection

*Pocillopora damicornis* colonies were collected from the eastern side of Coconut Island at the Hawaii Institute of Marine Biology, Oahu, Hawaii, United States of America under Hawaii Department of Land and Natural Resource permit number SAP2005-35. Colonies were fragmented into nubbins, and maintained in flow-through raceways at least 45 days before the experiment. In the raceways, corals were exposed to peak planar incident irradiance of 372 µmoles of photosynthetic active radiation (PAR) photons/meter^2^/second of shadowed sunlight. Light measurements were determined using a Li-Cor quantum radiometer and photometer (LI-250A; Li-Cor, Lincoln, Nebraska, United States of America) with a planar incident sensor.

### Experimental treatments

To assess the responses of *P. damicornis* to heat stress, light stress, and their combinations, coral nubbins were subjected to six treatments for two experimental periods, a short term 24–48 hour period and a longer term of up to 127 hours. The treatments began at 06:00 a.m. after 8 hours of darkness, and were maintained for 10 hours under experimental parameters (Table 1): (1) nubbins were maintained under darkness for 10 hours (18 hours total) at 25°C; (2) nubbins were maintained under darkness for 10 hours at 32°C (following a night under a reference temperature of 25°C, resulting in 18 hours of darkness); (3) reference low light treatment (438 µmoles/meter^2^/second PAR peak natural irradiance using a neutral density filter) at 25°C; (4) low light at 32°C; (5) high light (2007 µmoles/meter^2^/second PAR peak natural irradiance) at 25°C; and (6) high light at 32°C. Treatments were conducted in triplicate in 37.9 L (10 gallon) aquaria that contained a recirculating pump to maintain constant water flow over the coral nubbins, and aquarium heaters and cooling reservoirs to maintain a constant set temperature. At the end of the experiment, fragments were collected from each replicate for morphological and biochemical assessment. The experiments were then extended and additional nubbins were maintained under the same experimental conditions for 127 hours and nubbins examined at the end of this period (Table 1). This resulted in nubbins being maintained in diurnal cycle (10 hours of light and 14 hours of darkness) of low light, high light or complete darkness (24 hours of darkness per day) at the two temperature regimes for up to five days. At 16:00 hour of each day, samples were collected from each treatment. A 75% change of the water was made in each aquarium at 49 hours and 96 hours after initiating experimental treatments. Salinity was determined using a VEE GEE portable refractometer, (A366ATC; VEE GEE Scientific, Incorporated, Kirkland, Washington, United States of America) and constant salinity was maintained by adding double-distilled, de-ionized water as necessary. Samples were either snap-frozen in a liquid nitrogen vapor shipper for DNA abasic site, hydroxynonenal, and protein carbonyl assays, then stored at −80°C until processing or they were submerged in a chemical fixative. The processing of other samples is described below.

**Table 1:** Algal density in coral nubbins maintained under six environmental treatments and sampled after 48(short term) and 127 hours (long term) after the initiation of the treatments.

	Dark 25°C	Dark 32°C	Low-light 25°C	Low-light 32°C	High-light 25°C	High-light 32°C
**48 Hours**	1.26±0.08***a***	1.20±0.66***a***	1.33±0.02***a***	0.52±0.01***c***	0.71±0.06***e***	0.32±0.01***c***
**127 Hours**	0.87±0.04***b***	0.66±0.04***b***	1.25±0.05***a***	0.00***d***	0.41±0.2***c***	0.00***d***

All treatments except dark treatments were maintained under natural dark/light cycles. Treatments include: (1) a reference irradiance treatment of 438 µmoles/meter^2^/second PAR peak natural irradiance using a neutral density filter (low light) at 25°C (reference temperature), (2) low light at 32°C, (3) high light (2007 µmoles/meter^2^/second PAR peak natural irradiance) at 25°C, (4) high light at 32°C, (5) dark at 25°C, and (6) dark at 32°C. Entries in the table give treatment means ± SE. Treatment values with different letters differed significantly at α = 0.05 using the Tukey's Honestly Signficant Difference test.

### Collection and purification of *Pocillopora damicornis* zooxanthellae

Coral tissue was removed from the skeleton using a jet of filtered artificial seawater from a dental Water Pik (Water Pik, Inc., Ft. Collins CO). Coral tissue and cells were disrupted using a loose fitted-Teflon/glass Dounce homogenizer set at a slow speed. The tissue slurry was centrifuged at 1,000 *g* for 10 minutes using a fixed-angle centrifuge and the supernatant was checked for zooxanthellae contamination. Once it was free of zooxanthellae, the supernatant was discarded. The pellet was resuspended by pipetting and inversion with a buffer containing 3 units/mL of lysozyme (L6876, Sigma-Aldrich Corporation, St. Louis, Missouri, United States of America) and 2 units/mL of α-amylase (10102814001; Roche Diagnostics Corporation, Indianapolis, Indiana, United States of America) in artificial seawater, and the slurry then incubated on a rocking platform for 15 minutes. A second solution containing 2 units/mL of Dispase (17105-041; Life Technologies Corporation, Grand Island, New York, United States of America) in artificial seawater was added to the slurry, and incubated on a rocking platform for an additional 15–25 minutes. When the coral homogenate lost observable mucous aggregates, it was centrifuged for 10 minutes at 6,000 *g* using a swinging-bucket centrifuge. The supernatant was discarded and the pellet was resuspended in artificial seawater, and centrifuged for 10 minutes at 6,000 *g* using a swinging-bucket centrifuge. This wash step was repeated once. The final washed pellet was re-suspended in artificial seawater, and layered onto a Percoll cushion consisting of 30% Percoll (v/v), 10 mM polyethylene glycol 8000, 1 mM FICOLL 400,000 and seawater. The zooxanthellae were collected, washed with artificial seawater as above, to remove Percoll residue, and pelleted by centrifugation. The supernatant was removed and the pellet frozen (−80°C) for subsequent analyses.

### Algal density

Zooxanthella density provides a quantitative expression of the intensity of coral bleaching. A surgical bone cutter was used to excise a portion (3–7 mm diameter, 4–7 mm sectional width) from each coral nubbin. Algal density in these fragments was determined using a modified Marsh method [31]. Briefly, aluminum foil was wrapped around each coral fragment to determine the corresponding surface area of the tissue. The foil was removed and placed on a CannonScan flatbed scanner (Canon USA, Melville, New York, United States of America) and scanned beside a surface area calibrant. The surface area of the aluminum foil and calibrant was estimated using IMAGE J software (U. S. National Institutes of Health, Bethesda, Maryland, United States of America, http://imagej.nih.gov/ij/, 1997–2012).

*Pocillopora damicornis* is a non-perforate species with tissue only covering the outer skeleton, making it possible to recover all the tissue by scraping it from the skeleton using a stainless steel micro-chisel. The tissue was placed in 5 mL of artificial seawater (salinity 38 ppt) containing 50 mg of lysozyme and 5 mg of α-amylase and incubated on rocking platform for 15 minutes. Dispase was added (25 mg/mL in artificial seawater) to the sample slurry and incubated for 15 minutes. This cocktail of enzymes digested the coral mucus matrix, which otherwise can trap zooxanthellae during centrifugation and cause an artifact. The samples were centrifuged in an Eppendorf swinging-bucket centrifuge at 4,000 *g* for 10 minutes. The supernatant was removed and the pellet resuspended in 1.5 mL of artificial seawater. Algal pellets were homogenized in buffer (10 mM Tris-HCl, pH 8.0) and a sub-sample stored for determining cell numbers. Purified zooxanthellae were counted using a modified Neubauer hemocytometer (Hausser Levy Counting Chamber, Hausser Scientific Company, Horsham, Pennsylvania, United States of America). Zooxanthella density was then expressed as cells/cm^2^.

### Pigment analysis

**Figure 1:**
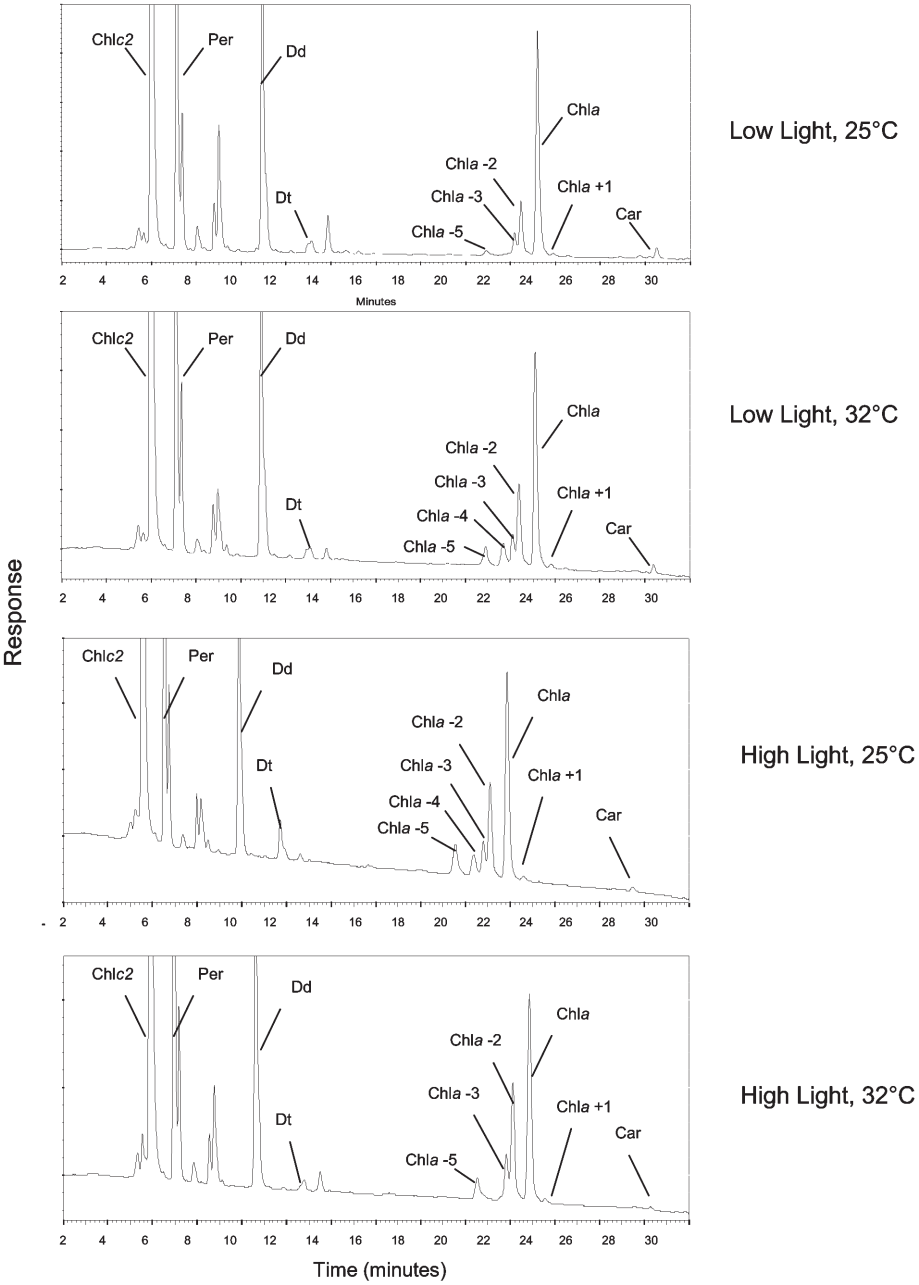
Representative chromatograms illustrating the peaks corresponding to the major pigments peaks denoted are chlorophyll *c*_2_ (chl *c*_2_) peridinin (per), cis-peridinin (cis-per), diadinoxanthin (Dd), diatoxanthin (Dt) and β-carotene (β-car) in dinoflagellates isolated from corals exposed to low light 25°C, low light 32C, high light 25°C, and high light, 32°C.

Dinoflagellate expulsion and digestion can both change the pigment profiles of corals. To assess this possibility, algal pigments were extracted from the remaining homogenate using methanol with the aid of ultrasonication. Extracts were clarified by centrifugation at 1800 rpm for 5 minutes, and the supernatant was drawn off for HPLC analysis. Aliquots of the clarified extract were vortexed with 1 M ammonium acetate solution (1∶1, v/v) and 100 µL injected onto a Hypersil- MOS2 C-8 column (3 µm particle size, 100×4.6 mm) maintained at 30°C. Pigments were separated using a binary mobile phase system (min; 70/30 (v/v) methanol/1.0 M ammonium acetate; 100% methanol): (0; 100; 0), (2; 50; 50), (12; 30; 70), (25; 10; 90), (27; 0; 100), (29; 0; 100), (29.1; 0; 100), (35; 0; 100). Flow rate was constant at 0.85 mL per minute. The analyses were performed using an integrated Thermo-Separations HPLC system comprised of a SCM1000 vacuum degasser, P4000 quaternary pump, AS3000 auto-sampler (with sample tray cooling, column over and sample preparation capability) and a UV6000LP diode array detector (DAD). System control, data collection and integration were performed using Chromquest software (Thermo Fisher Scientific, Incorporated, Pittsburgh, Pennsylvania, United States of America). Pigment identity was confirmed by co-elution with authentic pigments obtained from either Sigma Chemical Co or from DHI, Denmark (Fig. 1). Confirmation of analyte identity was achieved through spectral comparison of DAD data to standards using Chromquest software. Pigment quantification was carried out at 444 nm. Pigment concentrations were calculated as picograms per cell (pg cell^−1^) using the algal cell counts obtained as described above.

### Transmission electron microscopy

To examine the ultrastructure of zooxanthellae and their cnidarian hosts, a single polyp was biopsied from each nubbin using a 2.5 mm stainless steel leather punch. For primary fixation, the sample was submerged in modified Karnovsky's fixative (2.5% gluteraldehyde, 2% paraformaldehyde in 0.1M cacodylate buffer (pH 7.2) for 30 minutes and then transferred to a solution of 2.5% gluteraldehyde in filtered sea water. Samples were fixed overnight in this secondary fixative. For transportation, gluteraldehyde-fixed samples were placed into 1% gluteraldehyde in cacodylate buffer at 4°C. Samples were shipped from Hawaii, United States of America to Tel Aviv, Israel for final processing and electron microscopy under CITES export permit number 06US11157679.

Once in Israel, samples were washed three times for 15 minutes each in 0.1 M cacodylate buffer (pH 7.2), adjusted with NaCl to seawater molarity and pH. The samples were then decalcified with 10% Na_2_EDTA, pH 7-5-8.0 by first replacing the cacodylate wash buffer with the 10% EDTA solution and rinsing for 15 minutes. This was replaced with a fresh EDTA solution every 12–24 hours until the tissue is decalcified. The samples were then post-fixed in 1% osmium tetroxide at 4°C for 30 minutes to enhance membrane preservation, then rinsed in buffer. Samples were dehydrated in a graded ethanol series, then in propylene oxide followed by gradual embedding in Araldite (502; Electron Microscopy Sciences, Fort Washington, Pennsylvania, United States of America). Samples in the final, full-strength araldite were subjected to a mild vacuum (400 mbar) for 1 hour at 25°C followed by overnight polymerization at 60°C. The resulting block was trimmed and 1 micron sections were cut and stained with toluidine blue [32], or the block was sectioned (60–90 nm) using an ultramicrotome and sections mounted onto 300 mesh copper grids. Ultrathin sections were stained with lead citrate. Sections through approximately the same mid-polyp body area were examined using a JOEL JEM-1230 at 80 kV transmission electron microscope and images photodocumented with a TVIPS TemCam-F214 (Tietz Video and Image Processing System, Germany).

### DNA abasic lesions

DNA abasic lesions (DNA AP sites) were used as an indicator of genetic damage. Tissues from the skeletons of frozen coral nubbins were removed using a sterile micro-chisel and ground into a fine powder consistency using a liquid nitrogen-chilled mortar and pestle. Approximately 50 µL of frozen, powdered sample was placed into a microcentrifuge tube. DNA was isolated according to the manufacturer's instructions using the Dojindo Get pureDNA kit-Cell, Tissue (GK03-20; Dojindo Molecular Technologies, Incorporated, Rockville, Maryland, United States of America) with one slight modification to address Maillard chemistry artifact. The frozen tissue powder was added to the kit's lysis buffer containing 10 mg of polyvinylpolypyrrolidone (PVPP) (77627; Sigma-Aldrich Corporation, St. Louis, Missouri, United States of America). The DNA concentration was measured using an Invitrogen/Molecular Probes Quant-iT™ DNA Assay Kit, Broad Range (Q33130; Life Technologies Corporation, Grand Island, New York, United States of America) using a Bio-Tek FL800 fluorescent microplate reader (BioTek Industries, Incorporated, Winooski, Vermont, United States of America). DNA AP (abasic site) sites were quantified using the Dojindo DNA Damage Quantification Kit-AP Site Counting (DK-02-10; Dojindo Molecular Technologies, Incorporated, Rockville, Maryland, United States of America) and conducted according to the manufacturer's instructions.

### Protein carbonyl

A modified method of Robinson et al. [33] was used to measure the concentration of protein carbonyl groups, which are biomarkers of oxidative stress, specifically the oxidation of proteins. Approximately 50 µL of frozen, powdered sample was placed into a microcentrifuge tube. A solution containing 50 mM Tris-HCl (pH 7.8), 0.1 mM α-tocopherol, 0.005 mM salicylic acid, 20 mM phenylmethylsulfonyl fluoride, 20 mM benzamide, 50 µM a-aminocaproic acid, 1% polyvinylpolypyrrolidone (wt/vol), 0.15 mM desferoximine methylate, 0.01 mM sorbitol, 1 mM MgCl_2_, and 2 units of deoxyribonuclease I from bovine pancreas (D 4527; Sigma-Aldrich 1 µg of protein/aliquot) was added to the sample to remove DNA, and to redox stabilize the sample. The sample was vortexed and incubated for 10 minutes in a 37°C water bath, with occasional vortexing. A 100 µL aliquot of a second solution containing 10% sodium dodecyl sulfate, 50 mM Tris-HCl (pH 7.8), 80 mM disodium ethylenediamine tetraacetic acid (pH 8) and 50 mM dithiothreitol was added to the sample as both a DNase stop solution and a general protein denaturing buffer. Samples were vortexed and incubated at 55°C in a water bath for 6 minutes with occasional vortexing. Samples were then centrifuged for 10 minutes at 10,000 *g*, and the supernatant transferred to a new microcentrifuge tube. DNA concentration was determined as described above for DNA abasic lesions, so as to ensure that DNA had been removed to avoid signal artifacts. Protein concentration was determined using a modified Ghosh method [34]. Oxidized bovine serum albumin standards for carbonyl concentration standards were a gift from Dr. Charles Robinson.

Samples were applied to Nunc 96-well MaxiSorp microplates and assayed in triplicate, with triplicate blanking of carbonyl reactivity using 20 mM sodium borohydride. The remaining steps follow the protocol of Robinson et al. [33] using a 0.2% solution of 2,4-dinitrophenylhydrazine (DNPH) as the derivatizing reagent. A solution of 5% casein (C8654; Sigma-Aldrich, w/v), 50 mM Tris-HCl (pH 7.8), 1 mM NaCl, 0.5 mM sorbitol, 0.15 mM desferoximine methylate, and 0.005 mM salicylic acid was used as a blocking solution instead of nonfat dry milk. DNPH was detected using a primary antibody, anti-dinitrophenyl mouse monoclonal IgG1 (gift from Dr. Charles Robinson), and a donkey anti-mouse Fab fragment conjugated with horseradish peroxidase (Jackson ImmunoResearch Laboratories, West Grove, Pennsylvania, United States of America) as the secondary antibody. NEN Western Lightning Plus (Perkin-Elmer, Waltham, Massachusetts, United States of America) luminal solution was used to generate a signal, which was read with a Bio-Tek FL800 series fluorescent/luminescent microplate reader.

### Hydroxynonenal adducted to protein

Lipid peroxidation can produce reactive aldehydes such as 4-hydroxynonenal. To quantify lipid peroxidation, ∼50 µL of frozen, powdered sample was added to a microcentrifuge tube, then 1 mL of the same solution used in the protein carbonyl assay, was added to the sample, except it substituted 5 mM butylhydrotoluene for the 1 mM MgCl_2_, and added 1 µg of ribonuclease A from bovine pancreas (R 6513; Sigma-Aldrich). The sample was incubated for 10 minutes in a 37°C water bath with occasional vortexing. A 100 µL aliquot of the general protein denaturing buffer used in the protein carbonyl assay was added to the sample and incubated at 65°C for 6 minutes with occasional vortexing. Protein concentration was determined using a modified Ghosh method [34]. The antibody against HNE adducted to protein was from Envirtue Biotechnologies, Incorporated (similar to US Biological cat# H6276-10), but was raised against an antigen of HNE adducted to bovine serum albumin and not keyhole limpet hemocyanin. The secondary antibody was a donkey anti-rabbit Fab fragment conjugated to horseradish peroxidase (111-036-047; Jackson ImmunoReseach). NEN Western Lightning Plus (Perkin-Elmer) luminal solution was used to generate a signal, which was read with a Bio-Tek FL800 series fluorescent/luminescent microplate reader.

Standards of HNE-adducted to protein were created by incubating 1 mg of HNE in 10 mL of 1 mM Tris-HCl (pH 8.5) with 1 mM of the following synthesized peptide (NH_2_-YFNDSQRQATKDAG-COOH) for one hour. Theoretically, HNE will form stable Michael addition-type adducts with lysine and histidine residues. The adducted peptide was lyophilized and then resolubilized in water and 1% SDS, and gel purified on a 20% acrylamide gel. The adducted peptide was electroeluted from the gel, and quantified using the Bicinchoninic Acid (BCA) method (B9643; Sigma-Aldrich) incubating the BCA with the sample at 60°C for 15 minutes. Absorbance was read at 562 nm using an OceanOptics USB4000 spectrophotometer (Ocean Optics, Incorporated, Dunedin, Florida, United States of America).

### Sample extraction for ELISAs targeted to zooxanthella proteins

About 50 µL of frozen zooxanthella pellet was placed in a locking 1.8 ml microcentrifuge tube along with 1.0 mL of a denaturing buffer consisting of 2% SDS, 50 mM Tris-HCl (pH 7.8), 15 mM dithiothreitol, 10 mM disodium EDTA, 5% polyvinylpolypyrrolidone (wt/vol), 0.05 mM sodium tetraborate, 0.005 mM salicylic acid, 0.001% (v/v) dimethyl sulfoxide, 0.01 mM AEBSF, 0.04 mM bestatin, 0.001 E-64, 2 mM phenylmethylsulfonyl fluoride, 2 mM benzamide, 5 µM a-amino-caproic acid, and 1 µg/100 uL pepstatin A. Samples were vortexed for 15 seconds, heated at 93°C for 6 minutes, with occasional vortexing, and then incubated at 25°C for 10 minutes. Samples were centrifuged at 13,500 *g* for 8–10 minutes) and the middle-phase of the supernatant was aspirated and placed into a new tube [34]. Protein concentration of the supernatant was determined by the modified Ghosh method [34].

### Primary antibody validation

Antibodies to algal heat shock protein 70 (Hsp70), algal glutathione peroxidase, algal manganese superoxide dismutase, and the chloroplast small heat-shock protein were gifts from EnVirtue Biotechnologies, Inc. These antibodies were raised against synthetic peptides derived from conserved, yet unique, domains within these target proteins that showed little or no homology (less than 30%) with their invertebrate counterparts. The antigen sequence for algal Hsp70 was NH_2_-FEVLSTSGDTHLGGDD-COOH; for algal glutathione peroxidase, NH_2_-AFPCNQFGGQEPG-COOH; and for algal manganese superoxide dismutase, NH_2_–PILGLDVWEHAYYLKYQNRRP-COOH. The antigen sequences for plant algal chloroplast small heat-shock protein were based on a modified design of a multiple antigenic peptide (MAP) anchored to a poly-lysine dendritic body [35]. The antigen sequences for plant algal chloroplast small heat-shock protein are NH_2_–SPMRTMKQML-COOH, NH_2_–RADMPGLSKEDVKVS-COOH, and NH_2_–SSYDRTLRLPD-COOH. Non-MAP synthetic peptides conjugated with ova albumin were used to immunize rabbits. Antibodies were affinity purified from the resulting antisera.

The primary antibodies for zooxanthella protein targets were validated using protein separation by SDS-polyacrylamide gel electrophoresis and western blot transfer of the gel contents onto a membrane for immunochemical detection. Total soluble protein (25 µg) from two prepared samples was electrophoresed in a 12.5% SDS-PAGE preparative gel until the bromophenol blue dye front was near the bottom of the gel. All gels were transferred onto 0.2 µm polyvinylidene fluoride (PVDF) membranes (Immobilion P, EMD Millipore Corporation, Billerica, Massachusetts, United States of America) using a wet transfer system. The membranes were blocked in 5% non-fat dry milk, Tris-buffered saline (pH 8.4) (TBS) solution, and incubated with primary antibody for 1 hour at 25°C. Blots were then washed in TBS four times, and incubated in a 1∶30,000 dilution of alkaline phosphatase conjugated goat anti-rabbit secondary antibody solution (Jackson ImmunoResearch Laboratories) for 1 hour at 25°C. Blots were washed again four times in TBS, and developed using a nitroblue tetrazolium/5-bromo-4-chloro-3-indolyl phosphate solution (B5655; Sigma-Aldrich). Results were documented using a CanonScan Lidi50. To ensure a minimum of non-specific cross-reactivity, blots were developed for at least 3 minutes (data not shown).

As a final confirmation that primary antibodies cross-reacted with the appropriate epitope, 1 mM of unconjugated peptide antigen (used to generate the respective primary antibody) was added to 1 µL of primary antibody neet serum in 20 mL TBS. The solution was incubated for 30 minutes on a rocking platform. Blots were then added to the solution after being blocked with milk for 1 hour (like the rest of the primary antibodies), and processed as the other blots.

### Enzyme linked immunosorbent assays (ELISAs)

About 50 µL of frozen sample powder was placed in locking 1.8 mL microcentrifuge tubes along with 1400 µL of a denaturing buffer consisting of 2% SDS, 50 mM Tris-HCl (pH 7.8), 15 mM dithiothreitol, 10 mM disodium EDTA, 3% polyvinylpolypyrrolidone (wt/vol), 0.005 mM salicylic acid, 0.001% (v/v) dimethyl sulfoxide, 0.01 mM AEBSF, 0.04 mM bestatin, 0.001 E-64, 2 mM phenylmethylsulfonyl fluoride, 2 mM benzamide, 5 µM a-amino-caproic acid, and 1 µg/100 µL pepstatin A. Samples were vortexed for 15 seconds, heated at 93°C for 6 minutes with occasional vortexing, and then incubated at 25°C for 10 minutes. The samples were centrifuged, the middle-phase of the supernatant aspirated, and protein concentrations determined as described above for ELISA assays.

Total soluble protein from a sample (25 ng) was added to a well of a Nunc MaxiSorp 96-well microplate and incubated for 12 hours in a humidified chamber at 25°C. The sample solutions from each well were aspirated using a Bio-Tek EL404 Microplate Autowasher. Wells were blocked for 1 hour using a 5% (w/v) solution of non-fat dry milk in Tris-buffered saline. The blocking buffer was removed and adsorbed proteins incubated with primary antibody for 1 hour at 25°C. Plates were next washed with Tris-buffered saline using the microplate autowasher, followed by the addition of the appropriate secondary antibody conjugated with horseradish peroxidase (Jackson ImmunoResearch Laboratories). Signals were generated and read as described for the protein carbonyl assay. All samples were assayed in triplicate, with intra-specific variation of less than 11% throughout the 96-wells of each microplate.

### Statistical analyses

Data were tested for normality using the Kolmogorov-Smirnov test (with Lilliefors' correction) and for equal variance using the Levene Median test [36]. A one-way analysis of variance followed by Dunnett's Procedure [36] was used to test the null hypothesis that algal and host responses did not differ significantly between the control treatment (low light at 25°C) and all others. This protocol simplified interpretation of quantitative results from the cellular-physiological assays along with qualitative results obtained from micrographs. When data did not meet the homogeneity of variances requirement for one-way ANOVA, we instead used a Kruskal-Wallis One-Way Analysis of Variance on Ranks. Responses after 48 and 96 hours were evaluated separately, and α were reduced to 0.025 to reflect that samples taken from the same coral nubbin were analyzed twice, once at each time point. To facilitate other hypothesis tests, figures include results of means comparisons using Tukey's Honestly Significant Difference method (α = 0.025). Pigment and ELISA parameters of all six treatments over four days of exposure were analyzed using a two-way ANOVA with Tukey's Honestly Significant Difference test with a Bonferonni adjustment, the α reduced to 0.01667. All analyses were performed using JMP Pro Version 9 or 10 (SAS Institute, Incorporated, Cary, North Carolina, United States of America).

## Results

### Gross level changes: short- and long-term temperature and light stress induced bleaching

Significantly reduced zooxanthella density, or chlorophyll levels as compared to normal, non-stressful conditions often identify coral bleaching. To verify that experimental stressors caused *P. damicornis* to bleach via loss of zooxanthellae, zooxanthella densities of the corals under the different stress treatments were compared to those under the reference treatment: low light and 25°C. Under this reference condition, dinoflagellate density did not change significantly between 48 and 127 hours of exposure (t = 1.5, 4 df, *p*>0.20), and averaged 1.29×10^6^±0.03 algal cells cm^−2^. In contrast, after a 48 hour exposure to 32°C in low light, zooxanthella density decreased significantly (>59%; one-way ANOVA F_5,12_ = 31.7, *p*<0.0001; Dunnett's Procedure for this comparison, *p*<0.0001) and by 127 hours of exposure to those conditions, coral nubbins were completely bleached and zooxanthellae were undetectable in the tissues (Table 1). Similarly, corals exposed to high light-levels at 25°C had significantly lower densities of zooxanthellae than the references at both two days (48 hours) and 5 days (127 hours) (47% and 67%, respectively, Dunnett's Procedure *p*<0.0001, Table 1) and corals exposed to the dual stressors of high temperature and high light for 48 hours had significantly lower (76%, Dunnett's Procedure *p*<0.0001) dinoflagellate densities than reference corals. By 127 hours, the coral nubbins had completely bleached and zooxanthellae were undetectable in the combined stressor treatment (Table 1).

Corals exposed to prolonged darkness will also bleach, but these experimental treatments did not last long enough to induce a significant progression of visualized bleaching at reference temperatures. Instead, the darkness treatments permit inferences about the mechanism of temperature-induced bleaching in the absence of light. Dinoflagellate densities in corals exposed to darkness for 48 hours at 25°C were not significantly different from the reference, though corals exposed to darkness for five days (127 hours) at this temperature had marginally lower densities of dinoflagellates compared to the reference colonies (31% lower; Dunnett's Procedure, *p* = 0.04). Similarly, dinoflagellate densities in corals exposed to darkness for 48 hours at 32°C were not significantly different from the reference (Dunnett's Procedure, *p* = 0.18), though dinoflagellate densities in corals exposed to prolonged darkness (five days) at 32°C were significantly lower than in the reference corals (47% lower; Dunnett's Procedure, *p*<0.003; Table 1).

### Responses of *Pocillopora damicornis* to short-term exposure to bleaching conditions

**Figure 2:**
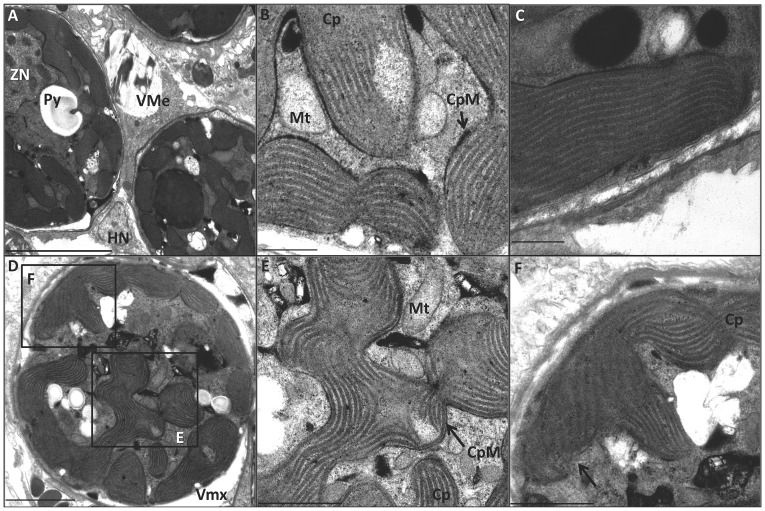
Transmission electron micrographs of a zooxanthella within the endodermal layer of the host coral *P. damicornis* exposed to 400 µµµµµmoles/meter^2^/second PAR peak irradiance (low-light) at 25°C and 32°C. Cp = chloroplast; CpM = chloroplast membrane; HN = host nucleus; MT = mitochondria; Py = pyrenoid body; T = thylakoids; VMe = host vacuolar membrane; Zn = Zooxanthella nucleus. (**A**) Low-light at 25°C (reference treatment). Magnification 1500×, Scale bar = 5000 nm. (**B**) Magnified area from boxed area in A. Magnification 8000×, scale bar = 500 nm. (**C**) Low-light at 25°C, magnified view of thylakoids in the chloroplast. Magnification 8000×, scale bar = 500 nm. (**D**) Low-light at 32°C. Magnification 2500×, scale bar = 2000 nm. (**E**) Magnified area from boxed area in D. Magnification 6000×, scale bar = 1000 nm. Arrow indicates intact trilaminate chloroplast membrane. (**F**) Magnified area from boxed area in D. Magnification 6000×, scale bar = 1000 nm. Arrow indicates diffused and disorganized chloroplast membrane zone.

Electron microscopy of reference samples (25°C, low light) revealed tight association of the vacuolar membrane with the dinoflagellate (Fig. 2A). Double membranes of the dinoflagellate mitochondria were distinct and coherent, as were the trilaminate membranes of the chloroplast (Fig. 2B). In most, the chloroplast thylakoids have parallel lamellar structures four membranes thick (Fig. 2C). However, some dinoflagellates were also observed with different condensed chromatin configurations and thylakoid lamellar arrangements with three or five membranes (data not shown), which supports Blank's argument [37]–[39] that cnidarians may host different species of dinoflagellates.

### Initial responses of *Pocillopora damicornis* to heat stress alone

Initial exposure to heat stress resulted in the dissolution of thylakoid membranes, accumulation of oxidative damage products, and induction of the stress protein response. Dinoflagellates in corals exposed to 32°C and low light were distinctly different from those found in the reference treatment. Most electron micrographs showed prominent vacuolization of the host tissue surrounding the algae (Fig. 2D). In many algal chloroplasts, a ‘dissolution’ or dispersion of lamellar thylakoids was observed (Fig. 2E, 2F). In some cases, plastid trilaminate envelopes were intact (Fig. 2E) but the plastid membrane was breached, and appeared as a blended zonation of both diffused chloroplast thylakoid membranes and stroma with the cytoplasm (Fig. 2F).

**Figure 3:**
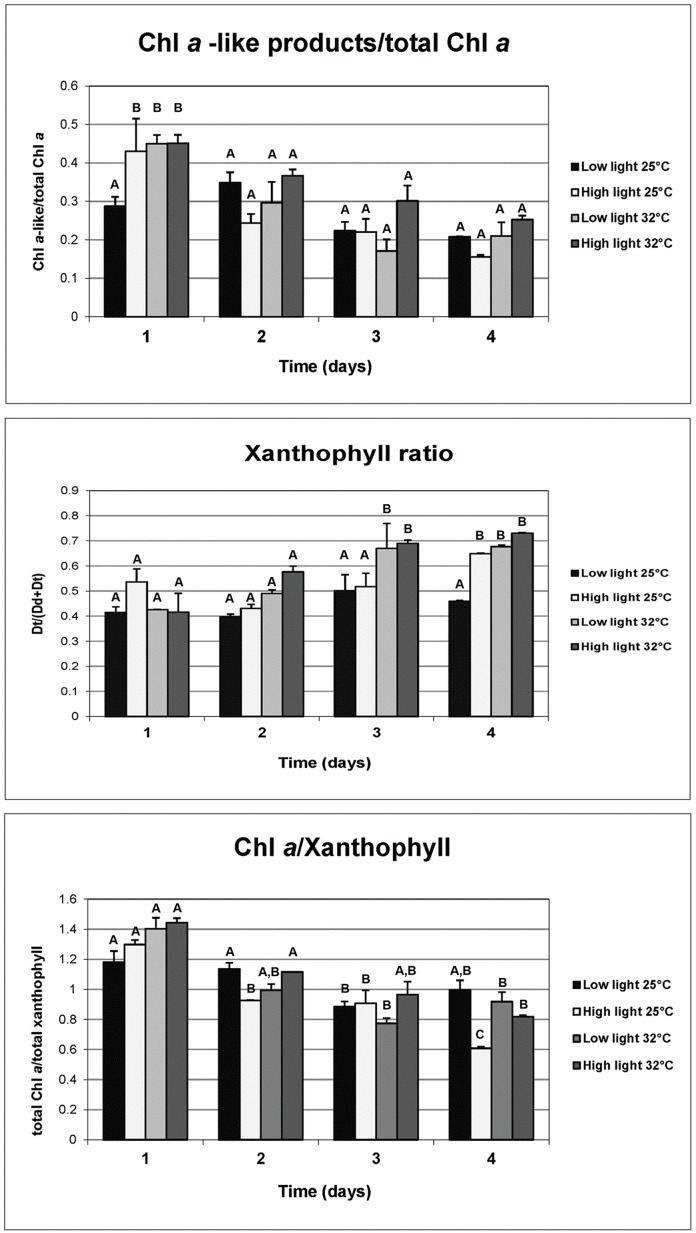
Concentration of major photosynthetic pigment ratios of zooxanthellae from the four light treatments collected from all four days of the experiment: (1) low-light at 25°C, (2) low-light at 32°C, (3) high-light at 25°C, and (4) high-light at 32°C. Entries in each graph give treatment untransformed means (±1 SE). Data were analyzed using two-way analysis of variance with a Holm-Sidak post hoc test. Treatment means with different letters differed significantly as α = 0.05.

**Figure 4:**
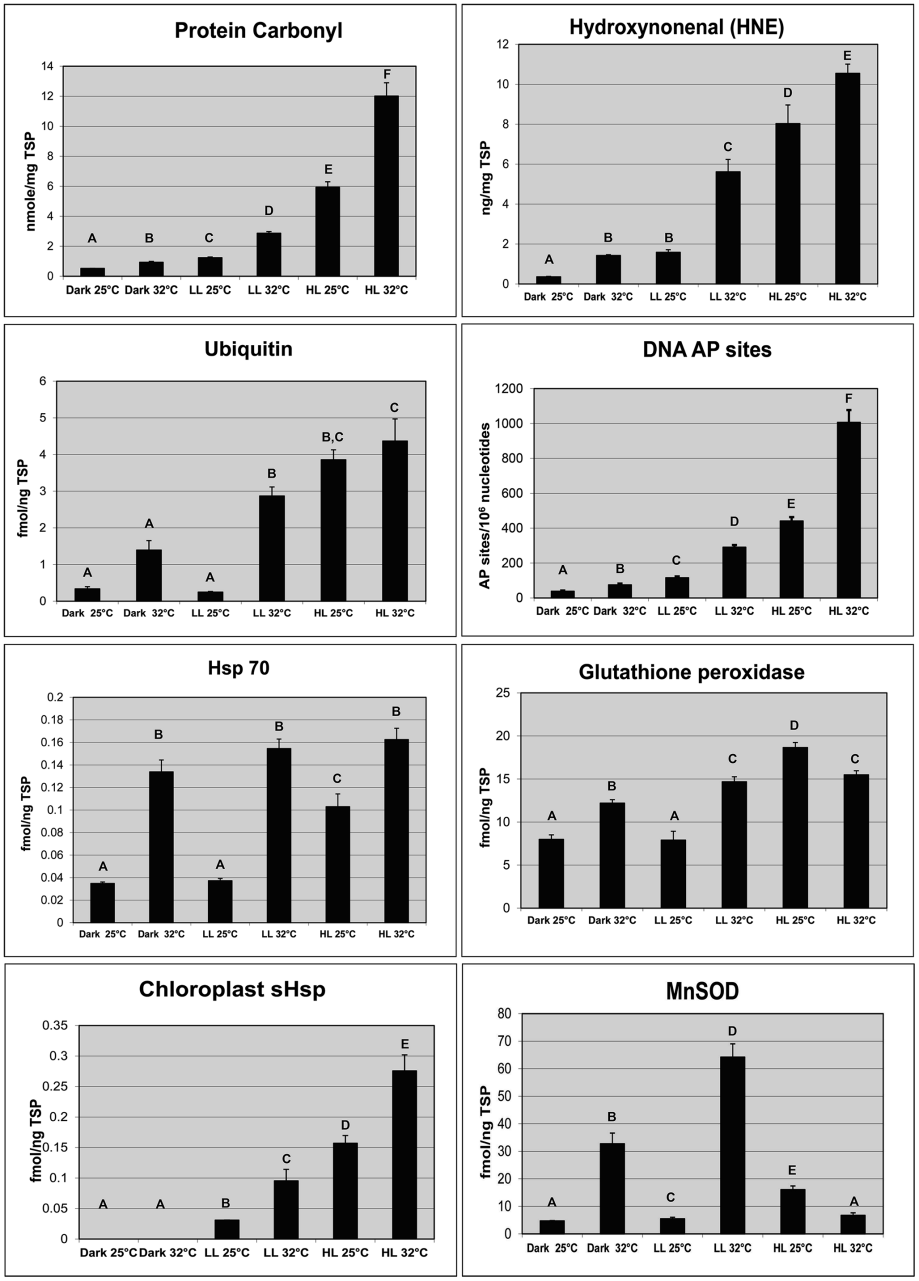
Biochemical parameters assayed from zooxanthellae isolated from corals exposed to the following six treatments and collected on Day 1 of the experiment: (1) dark, 25°C; (2) dark, 32°C; (3) low-light, 25°C; (4) low-light, 32°C; (5) high-light, 25°C; (6) high-light, 32°C. Entries in each graph give treatment untransformed means (±1 SE) and treatment bar means with different letters differed significantly at α = 0.025 using the Dunnett's Procedure.

**Figure 5:**
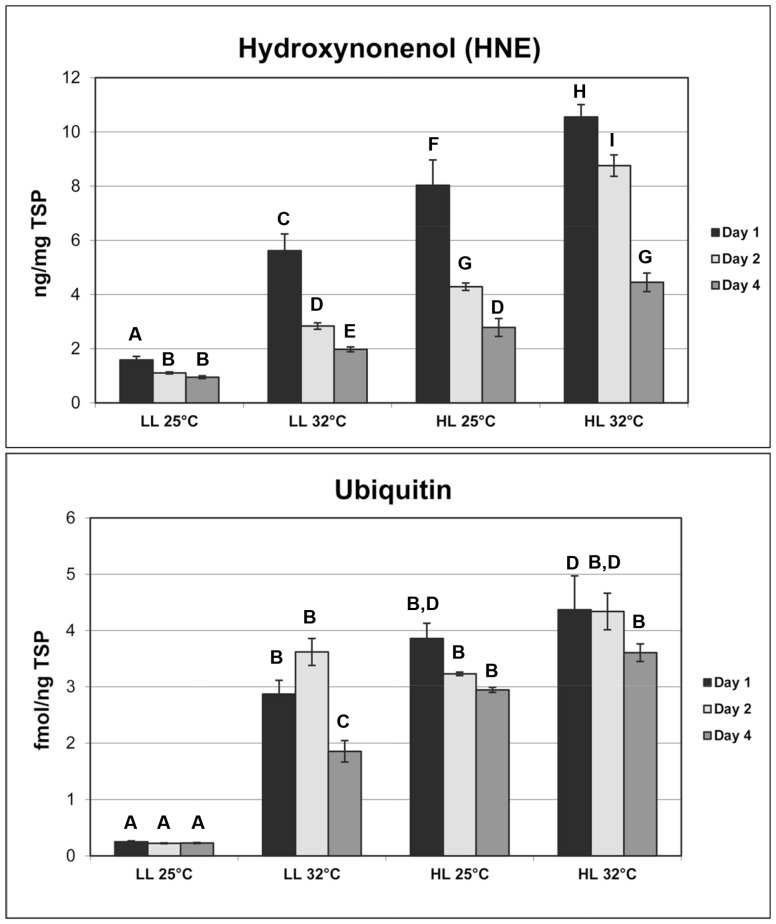
Hydroxynonenal and ubiquitin assayed from isolated zooxanthellae from corals exposed to the following six treatments and collected on Day 1, Day 2, and Day 4 of the experiment: (1) dark, 25°C; (2) dark, 32°C; (3) low-light, 25°C; (4) low-light, 32°C; (5) high-light, 25°C; (6) high-light, 32°C. Entries in each graph give treatment untransformed means (±1 SE). Data were analyzed using two-way analysis of variance with a Dunnett's Procedure. Treatment means with different letters differed significantly as α = 0.025.

Accompanying the morphological changes there was an accumulation of the chlorophyll *a* trans-product peak 4 (Figs. 1 and 3, Figs. S1 and S2) and an accumulation of oxidative damage products and induction of stress proteins were evident (Fig. 4). One form of biochemical lesions from oxidative damage (4-hydroxynonenal, Fig. 4) was a 2.5 fold higher in dinoflagellates exposed to low light at 32°C compared to the those from the reference treatment (one-way ANOVA F_3,8_ = 226.1, *p*<0.0001; Dunnett's Procedure for this comparison *p* = 0.0013). Two biomarkers of protein metabolic condition (ubiquitin and Hsp70) also were significantly elevated, when compared to the reference treatment (16.22X and 11.7X, respectively; Fig. 4). These statistically significant differences (Welch ANOVAs F>4.75, *p*<0.0008; Dunnett's Procedure for these comparisons, both *p*<0.0001) in mean expression levels indicate algae experienced extensive protein denaturation and increased protein degradation, likely from protein oxidation and adduction with aldehyde products [34] (Fig. 4; Fig. 5). Levels of the chloroplast small heat-shock protein (sHsp) were significantly higher in the low-light/32°C compared to the reference (Fig. 4), indicating a stress on Photosystem II, specifically the oxygen evolving complex. A significant accumulation (both F>36.4, *p*<0.0008) of the antioxidants glutathione peroxidase (GPx) and mitochondrial manganese superoxide dismutase (MnSOD) was also observed in response to heat stress (Fig. 4). Mean concentrations were 86% higher in this treatment compared to the reference for GPx, and 11.7 fold higher for MnSOD (Dunnett's Procedures for these comparisons, both *p*<0.0001).

### Initial responses of *Pocillopora damicornis* to light stress alone

**Figure 6:**
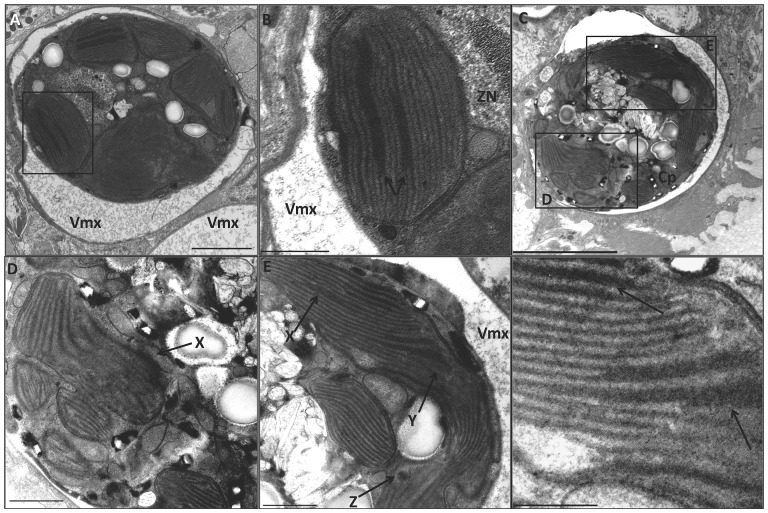
Transmission electron micrographs of a zooxanthella within the endodermal layer of the host coral *P. damicornis* exposed to 2007 µµµµµmoles/meter^2^/second PAR peak irradiance (high-light) at 25°C and 32°C. Cp = chloroplast; MT = mitochondria; s = starch granule; VMx = Vacuolar matrix; Zn = Zooxanthella nucleus. (**A**) High-light at 25°C. Magnification 2000×, scale bar = 2000 nm. (**B**) Magnified area from boxed area in panel A. Magnification 5000×, scale bar = 1000 nm. Arrow indicates coalescence of thylakoid membranes. (**C**) High-light at 32°C. Magnification 2000×, scale bar = 2000 nm. (**D**) Magnified area from boxed area in C. Magnification 4000×, scale bar = 1000 nm. Arrow indicates diffused and disorganized chloroplast membrane zone. (**E**) Magnified area from panel C. Magnification 4000×, scale bar = 1000 nm. **X**-arrow indicates coalescence of thylakoid membranes. **Y**-arrow indicates diffusion of thylakoid membranes into the stroma. Z-arrow indicates inverted thylakoid membrane micelle. (**F**) Magnified area from box in panel E. Magnification 12000×, scale bar = 500 nm. Arrows indicate coalescence of thylakoid membranes.

Initial exposure to light stress alone resulted in condensed columnated thylakoid membranes, accumulation of oxidative damage products, and induction of the stress protein response. Exposure to high light at 25°C produced significant vacuolization of host tissue around the dinoflagellate (Fig. 6A–B). Outer envelope membranes of both the algal plastid and mitochondria were intact (Fig. 6B). The most prominent feature resulting from light stress was condensation or fusion of multiple thylakoid lamellae. In contrast to the “heat stress alone” treatments, at high light alone, dissolution of lamellated thylakoids (Fig. 6B) was not observed.

Oxidative damage markers were significantly higher in high-light treatments compared to those measured in the heat only-stress and reference treatments, indicating a photo-oxidative stress (Fig. 4). Mean concentrations of protein carbonyl were 4.8 fold higher and mean HNE concentrations were 3.8 fold higher in the high-light/25°C treatment than in the reference treatment (Dunnett's Procedures for these comparisons, both *p*<0.0001). The mean number of DNA lesions nearly tripled in the high-light/25°C treatment compared to the reference treatment (Fig. 4; Dunnett's Procedure, *p*<0.0001).

Protein damage products also accumulated under these conditions. Mean ubiquitin concentrations were 14.5 fold higher than in the reference (Fig. 4; Dunnett's Procedure, *p*<0.0001). Antioxidant levels increased; mean glutathione peroxidase concentrations also were significantly higher than those in reference and heat-only stressed samples (Fig. 4; Dunnett's Procedure, p<0.0001), supporting the argument that light stress can drive oxidative stress. Mitochondrial MnSOD levels, though significantly higher than in the reference, were almost two-fold lower than levels seen in low-light/32°C samples, suggesting that the oxidative damage and perhaps the anti-oxidant response were specifically focused on the chloroplast (Fig. 4). Accumulations of chloroplast sHsp also more than doubled in the high-light/25°C treatment compared to the reference treatment (Dunnett's Procedure, *p*<0.0001). As in the heat-stress alone treatment, no photosynthetic pigments were significantly altered in response to light stress except chlorophyll *a* trans-product peak 4 (Figs. S1 and S2).

### Initial responses of *Pocillopora damicornis* to heat and light stress combined

Initial exposure to combined heat and light stress induced the unique thylakoid pathomorphologies observed during each stress alone, plus accumulation of oxidative damage products and induction of stress protein responses. Corals simultaneously exposed to high-light (2007 µmoles/meter^2^/second PAR peak natural irradiance) and temperature (32°C) exhibited extensive vacuolization around the dinoflagellates (Fig. 6C). In addition, algal chloroplasts often displayed breached plastid membranes (Fig. 6D), similar to those observed in the low-light/heat-stress treatments (Fig. 2E). Mitochondria were also observed to have breached membrane integrity, with a zonation of mixing with the mitochondrial matrix, mitochondrial membranes, and the cytoplasm (Fig. 6D). Thylakoid membranes showed pathomorphologies induced by both heat stress and light stress. Several chloroplasts displayed dispersion of thylakoid membranes (Fig. 6D); some chloroplasts had condensed thylakoid lamellae, while others had both condensed lamellae and dispersed lamellae (Fig. 6E–F). Inverted biphasic thylakoid micelles were also seen in chloroplasts of affected zooxanthellae (Fig. 6E; arrow z).

Concentrations of oxidative damage products were higher in the combined heat plus light stress treatments than in any others (Fig. 4). Mean concentrations of HNE and protein carbonyl were 2–3 times higher in the combined heat plus light stress treatment than in the next highest treatment, high-light at 25°C. The mean number of DNA abasic sites also were significantly more numerous than in all other treatments; lesions more than doubled compared to the next highest accumulation level (high-light/25°C; Fig. 4). As in the low-light/32°C treatment, the mean concentration of ubiquitin was significantly higher than the reference treatment (Dunnett's Procedure, *p*<0.0001), indicating a significant shift in protein metabolic equilibrium (Fig. 4). Concentrations of chloroplast sHsp were almost twice those in the high-light/25°C treatment, reflecting increased occurrence of both thylakoid pathomorphologies (Figs. 4 and 6C). Glutathione peroxidase concentrations were almost double those observed in the reference (Dunnett's Procedure, *p*<0.0001), but lower than in the high-light/25°C treatment. Mean concentrations of mitochondrial MnSOD were indistinguishable from the reference (Dunnett's Procedure, *p* = 0.9967; Fig. 4). Initial changes in photosynthetic pigments were observed in high light and light/temperature combinations (Figs. 1 and 3; Figs. S1 and S3). We did not observe any significant changes in photosynthetic pigments (Figs. S1 and S3).

### Responses of *Pocillopora damicornis* to prolonged darkness

**Figure 7:**
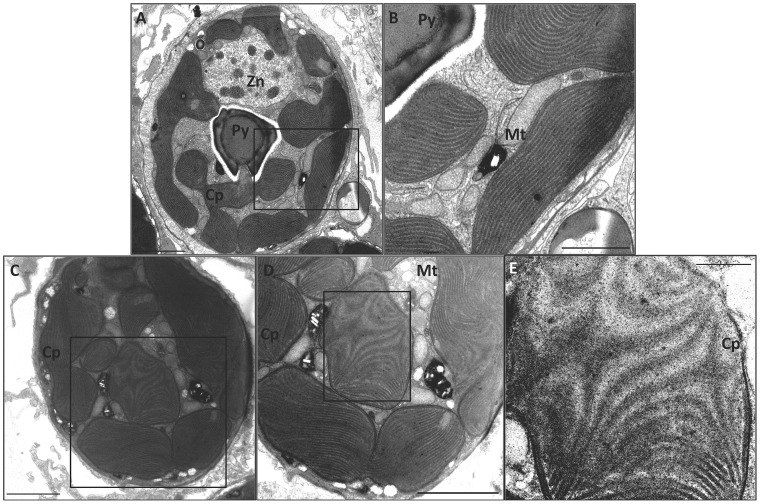
Transmission electron micrographs of a zooxanthella within the endodermal layer of the host coral *P. damicornis* exposed to darkness at 25°C and 32°C. Cp = chloroplast; Mt = mitochondria; Py = pyrenoid body; Zn = Zooxanthella nucleus. (**A**) Darkness at 25°C. Magnification 2000×, scale bar = 2000 nm. (**B**) Magnified area from boxed area in A. Magnification 5000×, scale bar = 1000 nm. (**C**) Darkness at 32°C. Magnification 2000×, scale bar = 2000 nm. (**D**) Magnified area from boxed area in C. Magnification 3000×, scale bar = 2000 nm. (**E**) Magnified area from boxed area in D. Darkness at 32°C. Magnification 8000×, scale bar = 500 nm.

To differentiate the effects of light and temperature on zooxanthellae, corals were exposed to 18 hours of darkness at 25°C and 32°C. Electron microscopy did not reveal pathomorphologies in dinoflagellates of corals maintained in prolonged darkness at 25°C, though slight vacuolization of host tissue around the zooxanthellae was observed (Fig. 7A–B). Prolonged darkness at 25°C and 32°C resulted in a significant reduction in oxidative damage marker levels compared with the reference (Fig. 4). This result was not unexpected because oxidative stress loads are predominantly driven by a photo-oxidative mechanism.

The initial exposure to darkness at 32°C caused dispersion of the thylakoid membranes. Prolonged darkness (18 hours) at 32°C resulted in vacuolization surrounding the algae and necrosis of gastrodermal cells containing the alga (Fig. 7A; *cf.* [30]). There were no signs of the chloroplast or mitochondrial outer membranes being breached (Fig. 7A–B). Thylakoid lamellae had extensive dispersion patterns, but there was no evidence of inverted micelles or thylakoid lamellar condensation (Fig. 7D–E).

In association with the morphological changes, there was a shift towards protein catabolism, and induction of anti-oxidant enzymes (Fig. 4). Mean concentrations of protein carbonyl were statically different among all the treatments (Student-Newman-Keuls Method; P = 0.005); the greater the stress load, the more protein oxidation products accumulated. Hydroxynonenal levels were significantly lower in coral exposed to 25°C darkness compared to all other treatments (Stuent-Newman-Keuls method; P = 0.007), and increased in accumulation in response to increasing stress conditions. Ubiquitin levels of the two dark treatments were not significantly different from the reference, though Hsp70 levels in dark/32°C were significantly higher than the reference, indicating a subtle shift in protein catabolism (Holm-Sidack method; P<0.001). Significant accumulations of both GPx and mitochondrial MnSOD (Dunnett's Procedure, both *p*<0.002) indicated that algae responded to an oxidative stress as a result of elevated temperatures. Mean concentrations of GPx were 1.5 fold higher, and mitochondrial MnSOD 6 fold higher, than in the low light/25°C reference treatment. No significant changes were observed in photosynthetic pigments (Figs. S1 and S3).

### Responses of *Pocillopora damicornis* to prolonged exposure to high light, high temperature, and combined stressors

To assess responses of coral to prolonged bleaching conditions, additional fragments were maintained under the same six experimental conditions for a period of five days. Corals exposed to treatments of low-light/32°C, high-light/25°C, and high-light/32°C lost over 60% of their algae after 48 hours, while corals exposed to low-light/25°C or the dark treatments showed no bleaching at 48 hours (Table 1). After 4 days of exposure though, significant mortality of corals was observed in both the low-light/32°C and high-light/32°C treatments. In contrast, dark-exposed corals bleached but did not die (Table 1).

In addition to pigment analysis, HNE and ubiquitin were the only biomarkers analyzed for the 48 hour (Day 2) and 96 hour (Day 4) samples, due to tissue limitations. After 2 days (48 hours) exposure, HNE levels were significantly lower in the low-light/32°C, the high-light/25°C and the high-light 32°C treatments compared to the levels in the same treatments at the first day of exposure. Levels were especially reduced in dinoflagellates from the high-light/25°C treatment (Fig. 5, P<0.01, F = 73.041, two-way ANOVA, Holm-Sidak post hoc test). Ubiquitin levels at 48 hours were not significantly different from those in their respective treatments at 24 hours (Fig. 5), though levels in all three treatments were significantly higher than reference levels at both 24 hours and 48 hours (Fig. 5, P<0.01, F = 44.493, two-way ANOVA Holm-Sidak post hoc test). The summed concentration of all chlorophyll *a*-like products was not significantly different among any of the treatments between day 1 and day 2 of the exposures, as were values for xanthophyll ratios and chlorophyll *a*/total xanthophyll ratios (Fig. 3). There were no significant differences in chlorophyll *a*, chlorophyll *c*_2_, or total chlorophyll *a* products among all treatments over the four days of the experiment (Fig. S3).

On Day 3 of the experiment, the ratio of chlorophyll *a*-like products to total chlorophyll *a* in the high-light/25°C and low-light/32°C was significantly lower than the levels they expressed on Day 1 (P<0.001, F = 8.603, two-way ANOVA, Holm-Sidak post hoc test), but these ratios were not significantly different from Day 1 and Day 3 reference values (Fig. 3). Xanthophyll ratios for the low-light/32°C and high-light/32°C treatments on Day 3 were significantly higher than all treatment ratios of Days 1–3 reference and high-light/25°C treatments (Fig. 3). Chlorophyll *a*/total xanthophylls ratios for the high-light/25°C, low-light/32°C, and high-light/32°C were significantly lower than for all treatment values on Day 1 (P<0.001, F = 6.244, two-way ANOVA, Holm-Sidak post hoc test), indicating a significant shift in the composition of the light harvesting complexes. There was insufficient sample material to assay ubiquitin and hydroxynonenal at this timepoint. On Day 4, HNE levels were significantly lower in the three stress treatments compared to the levels seen on Day 1 and Day 2 (P<0.001; two-way ANOVA, Tukey's Honestly Significant Difference test with a Bonferonni adjustment). Ubiquitin levels were also significantly lower in the three stress treatments compared to their levels exhibited on Day 2, though still higher than the reference levels of all the other exposure days (P<0.001; two-way ANOVA, Tukey's Honestly Significant Difference test with a Bonferonni adjustment; Figs. 4–5). Chlorophyll *a*-like products for the three stress treatments were not significantly different from those in the reference treatment on Day 1, or in the Day 4 reference (Fig. 3). Xanthophyll ratios in all three stress treatments were significantly higher than in the Day 4 references, and all treatments of Day 1 (Fig. 3; P<0.001; two-way ANOVA, Tukey's Honestly Significant Difference test with a Bonferonni adjustment). Chlorophyll *a*-like products/total chlorophyll *a* ratios for the high-light/25°C, low-light/32°C, and high-light/32°C were significantly lower than all treatment values on Day 1, while the ratios in the high-light/25°C were significantly lower than in the other three treatments for this day (Fig. 3; P<0.001; two-way ANOVA, Tukey's Honestly Significant Difference test with a Bonferonni adjustment).

## Discussion

Bleaching is a host-driven response that acts on the symbiont via either symbiophagy or expulsion [30]. One hypothesis is that bleaching is initiated by host-specific mechanisms independent of any influence from the dinoflagellate (e.g., heat-stress induced xenophagy; [30], [40]–[41]). An alternative hypothesis is that altered algal physiology initiates changes in the symbiotic equilibrium [20], [27]–[29]. Numerous studies correlate reduced Photosystem II efficiency and oxidative stress with heat- and light-induced coral bleaching [23]–[24], [26]. Some studies have correlated oxidative stress and heat- and light-induced bleaching, and proposed that deficient Photosystem II activity may be the primary source of oxidative stress, both in the dinoflagellate and host [23]–[24], [26], [42]. Despite advances in understanding the mechanisms of coral bleaching, difficulties remain in assessing causation, particularly in (1) determining the role and time-frame in which temperature and light act during a bleaching event, and (2) their relationship to other environmental stressors that ultimately induce bleaching [23], [29], [43]. Here, we provide evidence that temperature and light induce different pathologies in the symbiotic dinoflagellate during the early onset of bleaching, indicating that these stressors act on the algae by different mechanisms. Furthermore, we provide evidence that the sources of oxidative stress and the final receptors of its damage differ when bleaching is induced by heat-stress compared to light-stress.

Heat stress, whether applied on corals maintained under low intensity light or prolonged darkness, induced dispersion of thylakoid membranes within the chloroplasts. Chloroplasts of vascular plants display few observable effects of thylakoid disorganization at 35°C, and structural deformities only appear around 45°C [44]–[46]. Plastid deformities in higher plants include swelling of the thylakoid lumen, phase separation of non-bilayer lipids and formation of galactolipid-enriched inverted micelles; none of which were observed with heat-stress in the low-light or darkness treatments [44]–[46]. Dinoflagellate chloroplasts lack grana, which in vascular plants have different lipid, protein, and isoprenoid compositions than unstacked thylakoids [47]. The long rows of lamellated thylakoids, as seen in symbiotic dinoflagellates, may arise from their unusual composition, and result in rather unique behaviors at temperatures considered non-stressful to thylakoids in higher-order algae and plants [47]–[48].

Our study indicates that thylakoid dispersion in these dinoflagellates directly resulted from increased temperature rather than oxidative stress because the same pathomorphologies appeared in the prolonged darkness/32°C treatment, where no substantive oxidative stress was apparent. One interpretation is that the oxidative stress observed in the low light/32°C treatment resulted from physical disruption of photosynthetic electron transport, which then induced photo-oxidative stress. In higher plants, heat stress (e.g., 32°–38°C) destabilizes the oxygen-evolving-complex of Photosystem II, thereby leading to acceptor-side photoinhibition [49]–[50], but it also causes physical dissociation of the light-harvesting complexes from the Photosystem II core complex [51]–[53]. Determining which pathway is the more thermolabile in symbiotic dinoflagellates may provide insight into the role of thylakoid membrane integrity in photoinhibition.

Light stress affected algae differently than heat stress, by condensing thylakoid lamellae and excluding the lumen. These changes could result from lipid auto-oxidation/fixation, where the by-products of lipid autoxidation, such as hydroxynonenals and aldehydes (e.g., malondialdhyde and formaldehyde) crosslink proteins, isoprenoids, and lipids, resulting in aggregate inclusions [54]–[55]. This in turn, adversely affects photosynthetic electron transport, and could explain why bleaching resulting from high-light stress correlates with decreased photosynthetic efficiency and increased damage to Photosystem II [23]–[24], [27], [55]–[56]. Oxidative stress was more pronounced in light-stress treatments compared to heat-stress treatments, likely because of photo-oxidative generation of a damaged photosynthetic electron transport chain [57]–[59]. Significantly greater accumulation of chloroplast sHsp in light-stressed samples compared to the low-light/32°C samples suggests increased stress on Photosystem II function [60]–[61], which is independent of heat stress. Significantly lower levels of mitochondrial MnSOD in the high-light/25°C treatment compared to the low-light/32°C treatment indicates that oxidative stress is not predominately in mitochondria. Greater accumulation of mitochondrial MnSOD in the low-light/32°C and dark/32°C treatments denotes that heat stress is causing a significant oxidative stress in the mitochondria, and perhaps in the cytosol, as indicated by elevated glutathione peroxidase levels.

Differing patterns of stress protein levels suggest that elevated temperature and light affect dinoflagellates via different mechanisms. For example, concentrations of Hsp70 were significantly lower at 25°C under both high and low light compared to 32°C, and concentrations of glutathione peroxidase were significantly higher in light-stressed corals regardless of the temperature. To differentiate between the effects of light and temperature on the symbiotic zooxanthellae, corals were exposed to prolonged darkness at both ambient and high temperatures. Electron microscopy did not reveal major pathomorphologies in the dinoflagellates of corals maintained in prolonged darkness at 25°C. In these samples, only slight vacuolization of the host tissue around the zooxanthellae was observed, while those in the 32°C treatment showed major changes. Furthermore, oxidative damage markers were significantly lower in dark-maintained corals from higher temperatures compared with the reference. These results were not unexpected because oxidative stress loads are predominantly driven by a photo-oxidative mechanism [62]. Our results strengthen the argument that heat and light stress differ in their modes of action: heat stress damages thylakoid structures prior to extensive oxidative damage, while in light-stress conditions, photo-oxidative stress is initially induced, resulting in structural damage to the chloroplasts.

From the tissue to the sub-cellular level, the sequence of events and the time scale over which they occur have been largely ignored in studies of coral bleaching. The best time-course of bleaching (natural or laboratory induced) reported to date is the annual solar bleaching event of the *Goniastrea aspera* intertidal reef in Ko Phuket, Thailand where initiation of bleaching to the recovery from bleaching was observed to occur over a 15 day period [1], [13]. Le Tissier and Brown [13] examined changes in zooxanthellae density, histology, photosynthetic efficiency, and photosynthetic pigments of corals before bleaching, during bleaching and through the recovery phase of a bleaching event. The onset of bleaching in the present study was much more acute than in the time course from Ko Phuket, but results of the two studies are consistent. Le Tissier and Brown [13] saw phenomena similar to that reported in this study; the symbiotic dinoflagellates were ultimately digested, as well as the peculiar behavior in chlorophyll *a* concentrations. However, they could not discern the nature of the dinoflagellate damage or the source of the damage, nor could they explain the unusual behavior of chlorophyll *a*. Our experiments demonstrate that even before morphological signs of plasma membrane and thecal plate degradation (occurring from symbiophagy) [30], the zooxanthellae incur tremendous internal damage as a direct result of either temperature or light stress. Electron micrographs of corals in the high-light/25°C treatment showed zooxanthellae exhibiting minor presentations of thylakoid and chloroplast membrane pathomorphologies without significant symbiophagic vacuolization. This pattern suggests that zooxanthella damage occurs before the onset of symbiophagy. Chlorophyll *a* levels did not differ significantly among any treatments over 4 days (Fig. S3) although the ratio of chlorophyll *a*-like compounds∶total chlorophyll *a* indicated that the large variance in total chlorophyll *a* levels resulted from accumulation of chlorophyll *a*-like products (Figs. S1 and S2) [63] and that over the bleaching event, zooxanthellae that were retained in the host must have expressed lower levels of these catabolites.

Our study provides evidence for a specific sequence of events that occurs during bleaching, regardless of whether the initiating stressor is heat, light, or the combination of both. Damage to zooxanthellae is a critical first step. This is characterized by ultra-structural damage to organelles, accumulation of biochemical lesions, and changes to metabolic efficiencies [13], [23]. One of the first homeostatic responses to cellular injury reported here and found across all taxa of life, is induction of stress proteins [64]. These proteins mitigate damage by stabilizing metabolic pathways and cellular structures [60],[65]. As time progresses beyond the initial shock, other homeostatic defenses come into play, particularly changes in the cell's redox capacity, *i.e.*, anti-oxidant enzymes, solutes, and isoprenoids. Changes in membrane composition and protein isoforms also can occur, enhancing the inherent stability of cellular structures and metabolic processes in the face of a persistent stress [66]–[68]. Recognizing the sequential nature of this process and characterizing these steps allows us to identify thresholds that determine whether a coral succumbs to environmental conditions conducive to bleaching or has sufficient homeostatic capacity to tolerate the stress event. For example, symbiotic dinoflagellate clades with an inherently greater tolerance to heat stress are known to have higher concentrations of specific unsaturated lipids, which provide increased lipid stability and fluidity [69]. Elucidating sub-cellular and cellular processes, such as molecular switches that activate symbiophagy or exocytosis, are necessary to differentiate between proximate mechanisms involved in physiological acclimation and those responsible for evolutionary adaptation. Understanding the role each of these processes play in a given bleaching response provides insight for predicting its severity, ultimate population effects, as well as the feasibility of potential mitigation options.

## Supporting Information

Figure S1 **Concentration of major photosynthetic pigments from zooxanthellae collected after the first day of exposure to the four light treatments: (1) low-light at 25°C, low light at 32°C, high light at 25°C, and high light at 32°C.** Entries in each graph give treatment untransformed means (±1 SE). There were no statistically significant differences among any of the treatments for any of the pigments.
(TIF)

Figure S2 **Concentration of major chlorophyll** ***a*** **-like products from zooxanthellae collected after the first day of exposure to the four light treatments: (1) low-light at 25°C, (2) low light at 32°C, (3) high light at 25°C, and (4) high light at 32°C.** Entries in each graph give treatment untransformed means (±1 SE). There were no statistically significant differences among any of the treatments for any of the pigments.
(TIF)

Figure S3 **Concentration of major photosynthetic pigments of zooxanthellae from all four light treatments collected from all four days of the experiment: (1) low-light at 25°C, low light at32°C, high light at 25°C, and high light at 32°C.** (1) low-light at 25°C, (2) low light at 32°C, (3) high light at 25°C, and (4) high light at 32°C. Entries in each graph give treatment untransformed means (±1 SE).
(TIF)
