# Supplementary figures and images for: Heat-Stress and Light-Stress Induce Different Cellular Pathologies in the Symbiotic Dinoflagellate during Coral Bleaching
Source: PLoS One. 2013 Dec 4;8(12):e77173. doi: 10.1371/journal.pone.0077173 (PMC3851020; doi:10.1371/journal.pone.0077173)

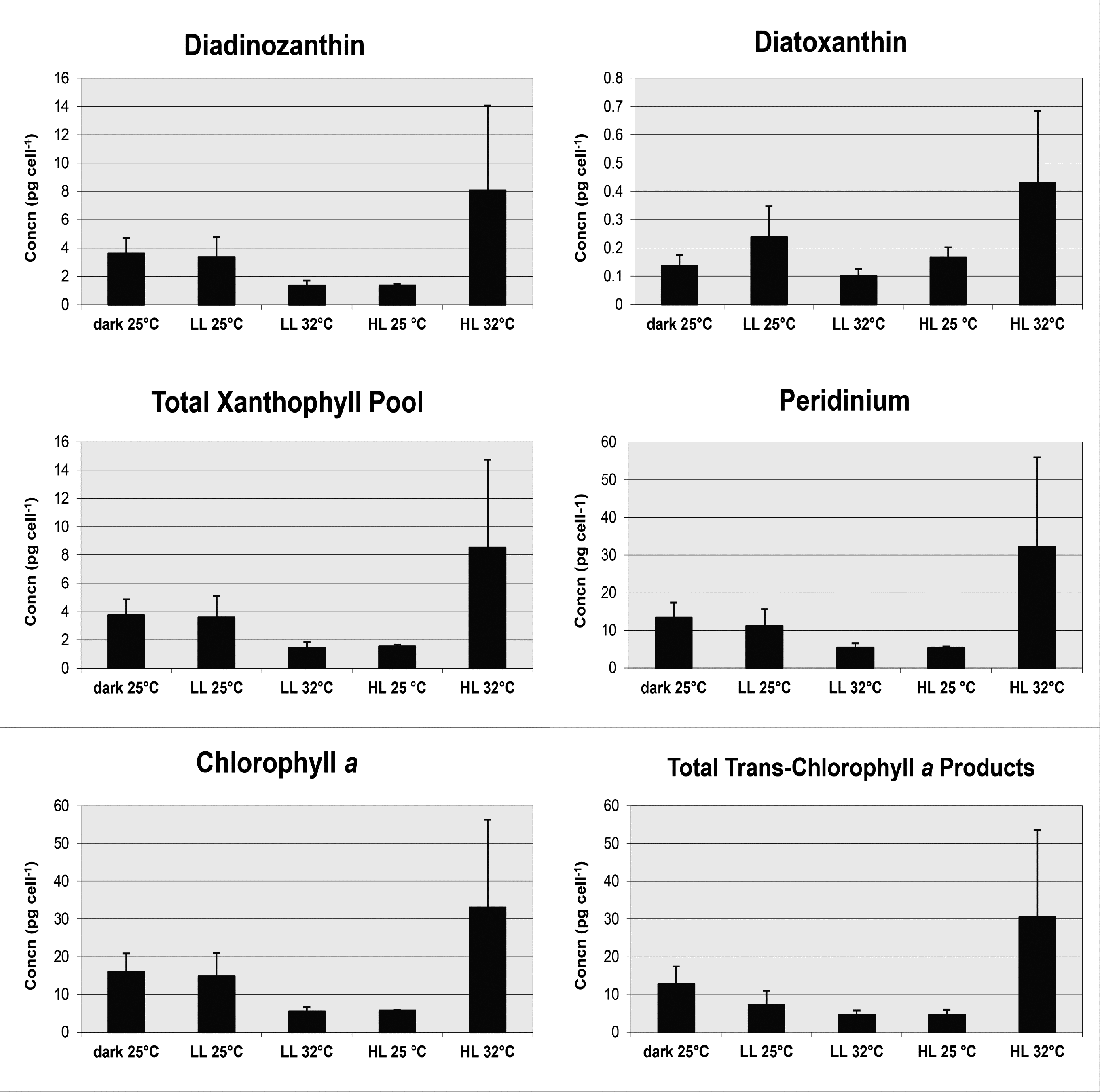

Supplement: Figure S1 — Concentration of major photosynthetic pigments from zooxanthellae collected after the first day of exposure to the four light treatments: (1) low-light at 25°C, low light at 32°C, high light at 25°C, and high light at 32°C. Entries in each graph give treatment untransformed means (±1 SE). There were no statistically significant differences among any of the treatments for any of the pigments. (TIF) [file pone.0077173.s001.tif]

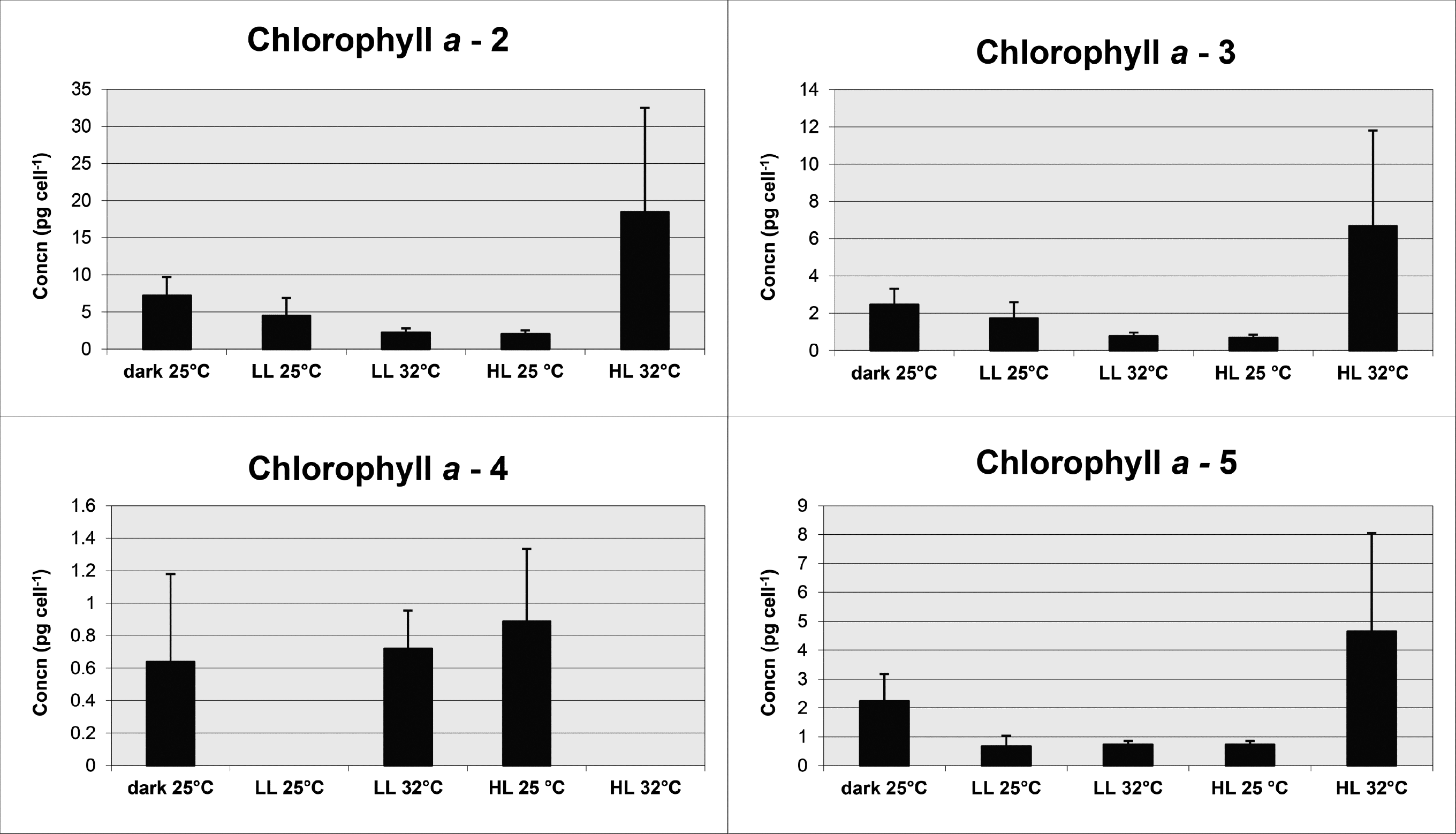

Supplement: Figure S2 — Concentration of major chlorophyll a -like products from zooxanthellae collected after the first day of exposure to the four light treatments: (1) low-light at 25°C, (2) low light at 32°C, (3) high light at 25°C, and (4) high light at 32°C. Entries in each graph give treatment untransformed means (±1 SE). There were no statistically significant differences among any of the treatments for any of the pigments. (TIF) [file pone.0077173.s002.tif]

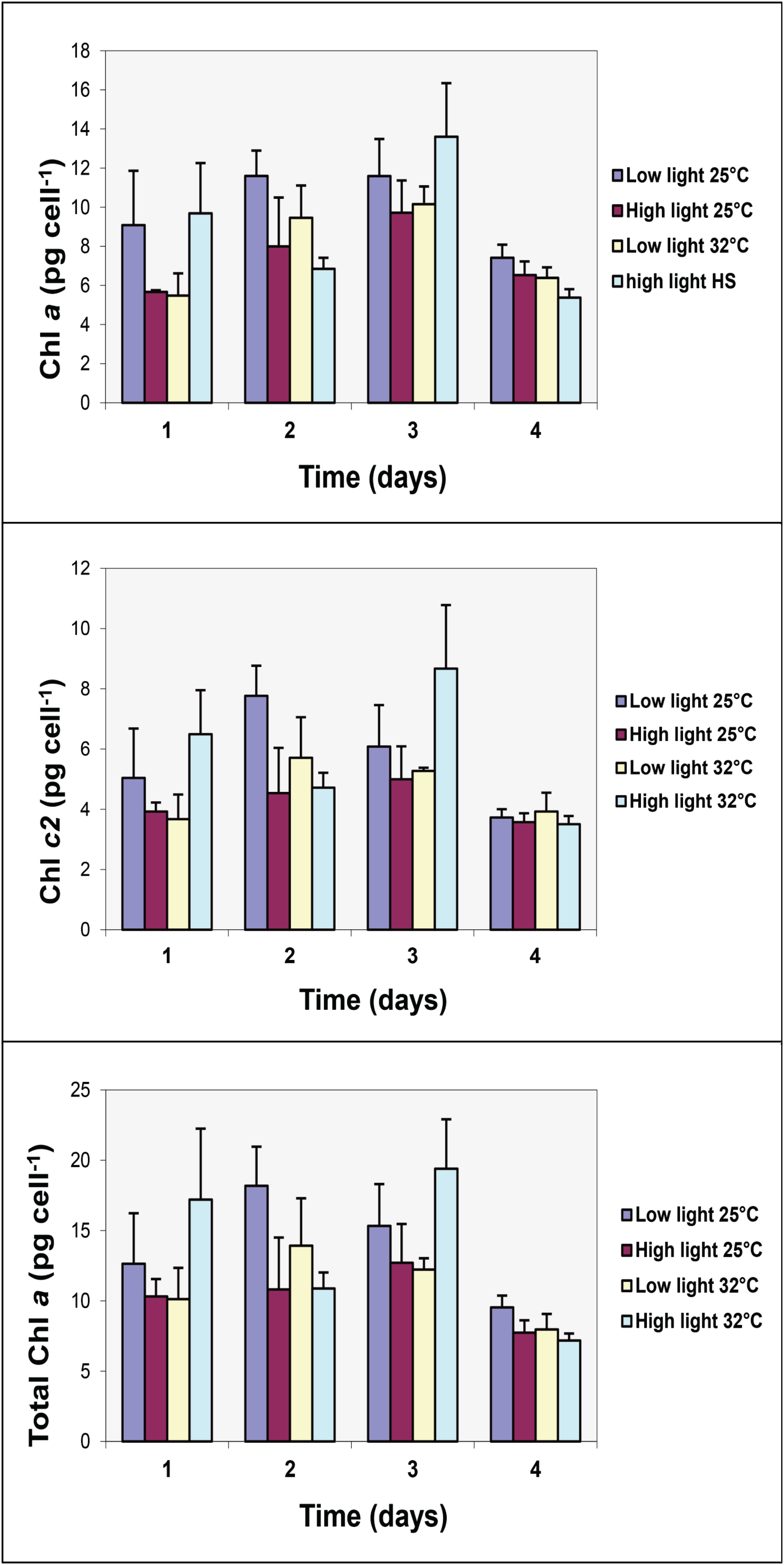

Supplement: Figure S3 — Concentration of major photosynthetic pigments of zooxanthellae from all four light treatments collected from all four days of the experiment: (1) low-light at 25°C, low light at32°C, high light at 25°C, and high light at 32°C. (1) low-light at 25°C, (2) low light at 32°C, (3) high light at 25°C, and (4) high light at 32°C. Entries in each graph give treatment untransformed means (±1 SE). (TIF) [file pone.0077173.s003.tif]
